# Heterogeneous Hybrid Nanocomposite Based on Chitosan/Magnesia Hybrid Films: Ecofriendly and Recyclable Solid Catalysts for Organic Reactions

**DOI:** 10.3390/polym13203583

**Published:** 2021-10-17

**Authors:** Metwally Madkour, Khaled D. Khalil, Fakhreia A. Al-Sagheer

**Affiliations:** 1 Chemistry Department, Faculty of Science, University of Kuwait, P.O. Box 5969, Safat 13060, Kuwait; Metwally.madkour@ku.edu.kw; 2 Chemistry Department, Faculty of Science, Cairo University, P.O. 12613, Gisa 12573, Egypt; kkhalil@taibahu.edu.sa; 3 Department of Chemistry, Faculty of Science, Taibah University, Yanbu 46423, Saudi Arabia

**Keywords:** chitosan, magnesia, heterogeneous catalyst, nanocomposites, Michael additions, Knoevenagel reactions

## Abstract

Chitosan/magnesia hybrid films (CS-Mg) have been prepared via sol-gel process and employed as heterogeneous catalysts. An in situ generation of a magnesia network in the chitosan matrix was performed through hydrolysis/condensation reactions of magnesium ethoxide. The synthesized hybrid films were characterized using various analytical techniques, such as X-ray photo-electron spectroscopy (XPS), field emission scanning electron microscopy (FESEM) and atomic force microscopy (AFM). The hybrid films display excellent catalytic activities in Michael and Knoevenagel reactions via one pot or solvent-free approaches under microwave irradiation conditions. Chitosan/magnesia hybrid films, catalysed pyrimidine, benzochromene, coumarin and arylidene-malononitriles derivatives formation reactions occurred with highly efficient yields of 97%, 92%, 86% and 95% respectively. Due to the fact that the films are durable and insoluble in common organic solvents, they were easily separated and can be recycled up to five times without a considerable loss of their catalytic activity.

## 1. Introduction

Over the past several decades, increasing attention has been directed toward the development of cleaner/greener synthetic methods, including processes that produce maximum yields of products, have minimum costs, avoid the use of toxic reagents and solvents, and proceed by synthetic routes that avoid the need for isolation of intermediates [1,2,3,4]. In this context, heterogeneous catalysts have advantages over their homogeneous counterparts because they can be easily isolated from reaction mixtures using simple filtration and can then be reused. As a result, reactions promoted by heterogeneous catalysts are economical and eco-friendly [5,6,7]. Magnesium oxide, a heterogeneous basic catalyst, has been recently used to promote several base-catalyzed organic transformations and is also an additive in refractory, paint and superconductor products [8,9,10,11]. As one of the most abundant natural biopolymers, chitosan (CS) possesses excellent properties, such as biocompatibility, biodegradability, non-toxicity, and insolubility in most solvents. Consequently, it has been used on its own or as part of films or fibers in several applications [12,13,14]. Chitosan has garnered significant interest, owing to the essential role it plays in transition metal catalyzed reactions [15]. For example, chitosan-supported metal complexes are used as catalysts for Suzuki cross-coupling (CS-supported Pb) [16], Henry (Cs-supported Ti catalyst) [17] and hydroformylation (CS-supported Rh catalyst) reactions [18]. Additionally, the biopolymeric nanocomposite (CS-Pr-Me-Cu(II)-Fe_3_O_4_) was investigated as a heterogenous catalyst for the oxidation of benzyl alcohols/Knoevenagel condensation [19]. Recently, we uncovered an efficient protocol for catalyzing Michael addition reactions that rely on the use of modified and non-modified chitosan as heterogeneous recyclable catalysts [4,5]. However, the major drawback of employing chitosan as a homogeneous catalyst is related to its propensity to form gels, which makes its separation and recovery difficult. The widespread use of heterocyclic compounds in agricultural, pharmaceutical, analytical, and medicinal chemistry areas has encouraged efforts aimed at the development of new methods to synthesize members of this wide family [20]. Molecules containing pyrimidine, coumarin, and benzopyran are highly important classes of N- and O-heterocycles that are widely used as valuable precursors for pharmaceutical agents [21,22]. Over the preceding decades, a considerable increase in devising microwave assisted organic processes has been apparent, due to their economic and green advantages [23]. 

In continuing efforts focused on the design of novel methods to prepare nanocomposites, which can be employed as heterogeneous catalyst, we have turned our attention to one pot or solvent-free approaches. Herein, we developed a method for the synthesis of pyrimidine, coumarin and chromene derivatives that rely on the use of CS-MgO nanocomposite films as heterogeneous catalysts for reactions carried out under microwave irradiation conditions. The CS-MgO hybrid nanocomposite films were prepared by incorporating magnesium ethoxide in the chitosan polymer matrix through use of the sol-gel process [24,25]. The morphology and size of the magnesia particles in the polymer matrix were determined using scanning electron microscopy. Studies of the utilization of the hybrid films as heterogeneous catalysts revealed that they serve as an eco-friendly recyclable catalyst and promote highly efficient Michael and Knoevenagel reactions.

## 2. Materials and Methods 

### 2.1. Materials

Medium molecular weight grade chitosan (deacetylated 90%, M = ca. 35.000), magnesium ethoxide (purity 98%) and nano-magnesium oxide powder (<50 nm particle size) were purchased from Aldrich (Sigma-Aldrich, St. Louis, MO, USA ). All other chemicals were of analytical grade and used without further purification. All products were characterized using FTIR (Varian 610-IR microscope system, Santa Clara, CA, USA), and ^1^H- and ^13^C-NMR. 

### 2.2. Preparation of the Nanocomposite Films

**Scheme 1:**
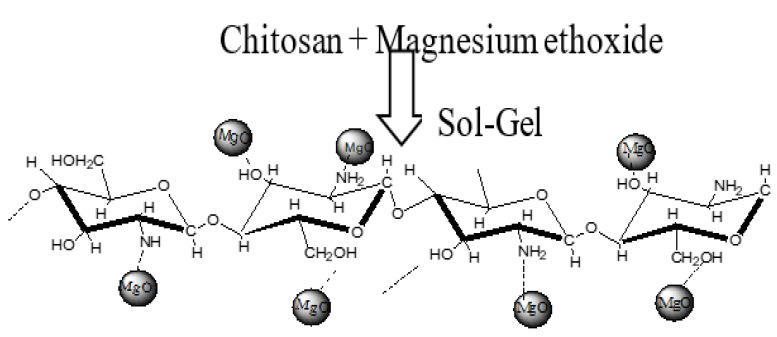
Chitosan-Magnesium nanoparticle hybrid film.

A solution of chitosan polymer (2 wt.%) was prepared by dissolving it in a 2% acetic acid aqueous solution. The prepared solution was permitted to stir for 48 h at room temperature to obtain homogeneous solution. A certain amount of this solution and a stochiometric amount of magnesium ethoxide were added to a 50 mL bottle (Scheme 1). The mixture was permitted to stir for 1 h at room temperature. A mixture of water and ethanol with volume ratio (1:1.5) was added to the solution and then the mixture was stirred for 18 h at room temperature to complete the sol-gel process. This solution was then casted in Teflon petri dishes and dried at 50 °C for 17 h. The films were then further dried under vacuum conditions for 48 h at 50 °C.

### 2.3. Characterization of the Chitosan-Mgo Hybrid Films

Field emission scanning electron microscopy (FESEM) (PhotoMetrics, Inc., Huntington Beach, CA, USA) was conducted to obtain insights into the shape and size of the prepared films using a (JEOL JSM-7001F). X-ray photoelectron spectroscopy (XPS) (Axis Ultra DLD Spectrometer, Kratos, Manchester, UK) was conducted to investigate the surface elemental composition of the hybrid films using a Thermo ESCALAB 250 Xi (Thermo Fisher Scientific, Waltham, MA, USA), using monochromatic Al Kα radiation (1486.6 eV) and a pass energy of 20 eV. The XPS deconvoluted spectra were conducted using Thermo Avantage software version v5.956 (Thermo Fisher Scientific) with Gauss-Lorentz maximum iterations of 100, a convergence of 0.0001 and a Powell fitting algorithm. All the binding energy values (BE/eV) were corrected with regard to the adventitious Carbon (C–H/C–C) for C1s at 284.6 eV. The surface topography was studied using a Nanoscope-IV multimode atomic force microscope (AFM) (Nanoscience Instruments, Inc., Phoenix, AZ, USA). A scan area of 5 μm was measured. The background was removed from the images using Nanoscope software. A thermogravimetry (TGA) (Mettler Toledo, Columbus, OH, USA) was performed with a 10 mg sample from ambient for up to 800°C at a heating rate of 10°C/min in air using a DTGA-60 Shimadzu (Shimadzu, Kyoto, Japan) automatic analyzer.

### 2.4. General Procedure for Synthesis of Pyrimidine Derivatives

A mixture of aldehyde (10 mmol), guanidine hydrochloride (0.95 g, 10 mmol), malononitrile or ethyl cyanoacetate (10 mmol) and CS-MgO nanocomposite films (5 and 10% MgO content) was dispersed in 10 mL of the selected solvent (See tables). The suspension was heated using microwave irradiation (100 W) for the appropriate amount of time (monitored by TLC (Silver Spring, Maryland) using 1:1 n-hexane/ethyl acetate as eluent). After completion, the film was removed from the hot mixture and was then washed thoroughly with hot methanol, using the simple Soxhlet method. The remaining mixture was then concentrated and poured into an ice/water mixture. The crude solid that formed was separated through filtration and then crystallized using hot ethanol. The previous method was repeated under the same employed conditions but in the presence of a catalytic amount of MgO nanoparticles (5 wt.%) in order to compare the potency of our nanocomposite to that of the MgO nanoparticles. 

### 2.5. General Procedure for Synthesis of Benzochromene Derivatives

A mixture of the aldehyde (10 mmol), 1-naphthol (1.44 g, 10 mmol), malononitrile (0.66 g, 10 mmol) and CS-MgO nanocomposite films (5 and 10% MgO content) was dispersed in 10 mL of the selected solvent. The mixture was heated using microwave irradiation (100 W) for the appropriate amount of time (monitored by TLC using 1:1 n-hexane/ethyl acetate as eluent). After the completion of the reaction, the film was removed, and the crude solid product was separated through filtration and crystallized from ethanol. Similarly, the previous method was repeated under the same employed conditions with the presence of MgO nanoparticles (5 wt.%) for comparison.

### 2.6. General Procedure for Synthesis of Coumarin Derivatives

A mixture of salicylaldehyde (1.22 g, 10 mmol), the active methylene compounds (10 mmol), and the CS-MgO nanocomposite film (5 and 10% MgO content) were mixed in 10 mL of the appropriate solvent. The mixture was heated using microwave irradiation (100 W) for the appropriate amount of time (monitored by TLC using 1:1 n-hexane/ethyl acetate as eluent). After the completion of the reaction, the film was removed, and the filtrate was concentrated. The residue was added to ice/water, producing a precipitate that was separated through filtration and crystallized from ethanol. This method was also repeated under the same employed conditions with the presence of MgO nanoparticles (5 wt.%) for comparison.

### 2.7. General Procedure for Synthesis of Heterocyclic Arylidene-Malononitriles

A mixture of furfural, thiophene-2-carboxaldehyde or pyridine-4-carboxaldehyde (10 mmol), malononitrile (0.66 g, 10 mmol) and CS-MgO nanocomposite films (5 and 10% MgO content) was heated using microwave irradiation (100 W) for 1 min. After the completion of the reaction, the film was removed through simple filtration. The crude solid product was separated by adding hot methanol followed by filtration and then crystallized from ethanol. The procedure was repeated under the same conditions but in the presence of MgO nanoparticles (5 wt.%) for comparison.

## 3. Results and Discussion

### 3.1. Characterization of Chitosan-Mgo Nanocomposite Films

#### 3.1.1. Microscopic Analysis

**Figure 1:**
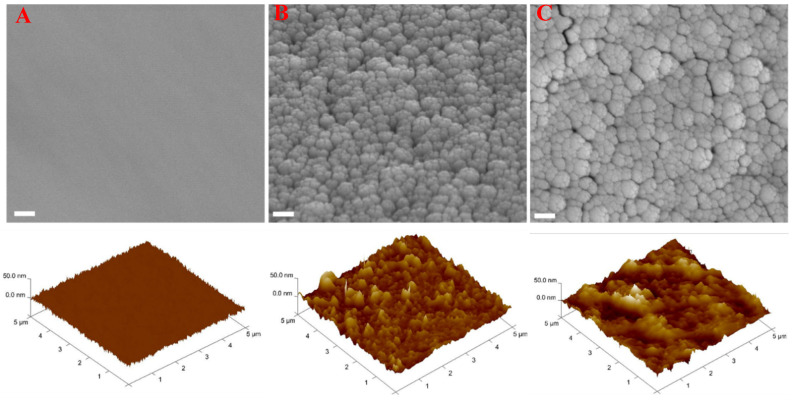
FESEM and AFM images of (**A**) Chitosan, (**B**) Chitosan-MgO 5 wt.% and (**C**) chitosan-MgO 10 wt.%. The scale bare for FESEM is 100 nm.

FESEM was used to assess the morphology and size distribution of the MgO nanoparticles in the polymer matrix. FESEM micrographs of pure chitosan (Figure 1A) and hybrid films containing magnesia particles of 5 wt.% (Figure 1B) and 10 wt.% are shown in Figure 1C. An inspection of the micrographs reveals that the surface of the pure chitosan is homogenous, continuous, and smooth. The MgO nanoparticles in the chitosan-MgO nanocomposite are visible in the form of clouds distributed in the polymer matrix. The nanoparticles appear as dense phases that resemble white spherical beads with somewhat dispersed surfaces. The blurred surfaces are a consequence of adsorption of the polymer layers on the particles surface. The images show that the inorganic phase is well dispersed in the chitosan matrix. The average size of the MgO nanoparticles in the 5 wt.% loading is approximately ~14 nm while it is ~11 nm for 10 wt.% loading which is comparable with the crystallite size of 10.7 nm calculated from XRD pattern (not shown). 

Furthermore, AFM was used to study the surface topology of the MgO/chitosan nanocomposite composite films and to assess the roughness and uniformity of dispersions of MgO in the polymer matrix. An inspection of the three-dimensional AFM images displayed in Figure 1 shows that the pure chitosan film has a smooth and flat textured surface indicating the absence of observable agglomerates (Figure 1A). In Figure 1B,C AFM images are shown from 5 wt.% and 10 wt.% of magnesia composite films, respectively. The surfaces of the hybrids have globular rough morphologies with some needles lying on the surface, most of which are oriented perpendicular to the surface. This observation indicates that the MgO nanoparticles are uniformly distributed in the chitosan matrix. As the magnesia content increases, the interactions of MgO with the polymer increases and the particles become well distributed and completely cover the polymer. The image of the hybrid is blurred because the densely distributed particles are engulfed by the polymer chains (Figure 1C).

#### 3.1.2. XPS Analysis

**Figure 2:**
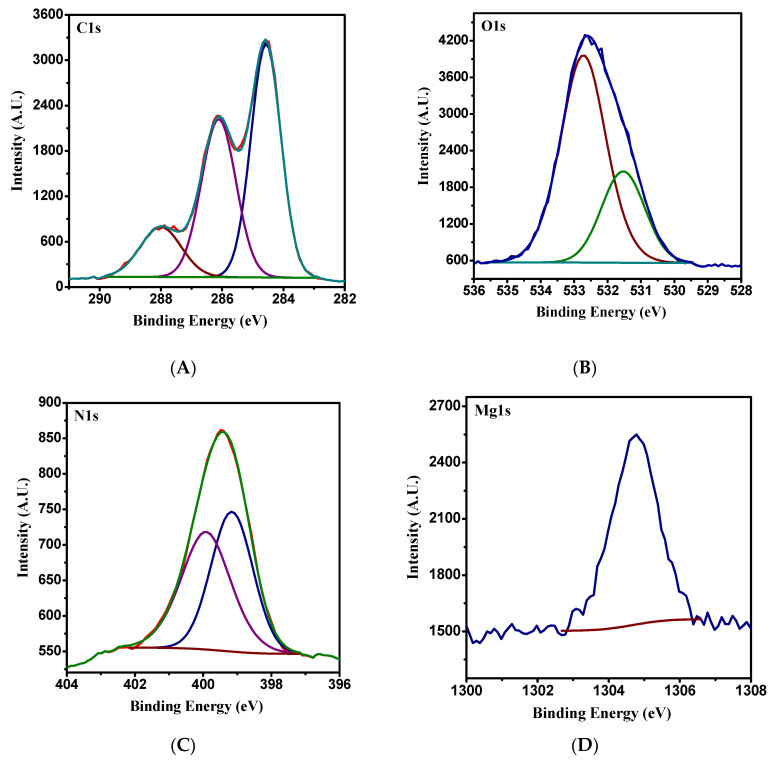
XPS binding energies 10 wt.% magnesia /chitosan hybrid film, spectra of C1s peak (**A**), O1s peak (**B**), N1s peak (**C**), Mg1s peak (**D**).

X-ray photoelectron spectroscopy (XPS) was utilized to investigate the surface elemental composition of the CS-MgO hybrid films. The binding energies of the elements in the chitosan/magnesia hybrid film, obtained from an analysis of the resolved XPS spectrum, for C1s (A), and O1s (B), N1s (C) and Mg1s (D) are provided in Figure 2. The peaks in the spectrum for C1s correspond to C–C/C–H (284.6 eV), C–O (286.2) and C = O (288.0 eV), the latter being attributed to acetyl groups on the chitosan backbone [26]. The results show that carbon, oxygen, nitrogen, and magnesium are present in the hybrid film. Two peaks exist in the O1s spectrum, and the peak at 531.5 eV is attributed to N–C–O chemical binding in *N*-acetylated-glucosamine units and the other peak, at 532.7 eV, is assigned to C–O, O–H or bound water [27,28]. The N1s XPs spectrum of the chitosan/magnesia nanocomposite film contains a peak at 399.1 eV that is attributed to the NH_2_ or NH groups and another at 400.0 eV is associated with the amine groups in the ammonium form (NH_3_^+^) [29]. The N peaks in the hybrid appear at lower binding energies compared to those of the pristine chitosan (399.1 and 400.0) [30], which indicates that the amine groups interact with MgO nanoparticles. The binding energy of Mg1s at 1304.5 eV is attributed to the Mg^2+^ in MgO nanoparticles [31]. 

#### 3.1.3. Thermogravimetric Analysis

**Figure 3:**
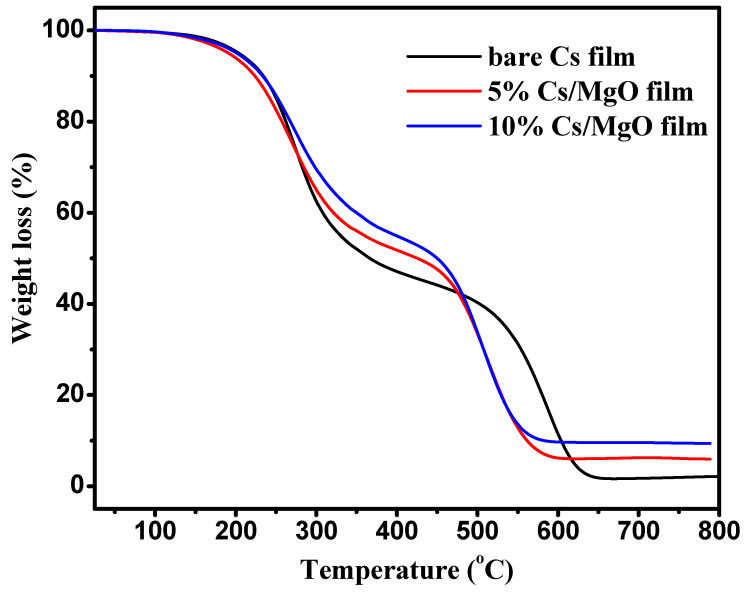
TGA of bare chitosan, 5 wt.% chitosan/MgO and 10 wt.% Chitosan-MgO films.

A thermal analysis was performed to assess the material‖s stability as a function of temperature to gain an understanding of their degradation behavior. The thermogravimetric analysis was performed using the sample under air in the temperature range of 30–800 °C. Figure 3 presents the weight loss versus temperature profile of pure chitosan (A) and of the hybrid materials with 5 wt.% (B) and 10 wt.% (C) of magnesia. The initial weight reduction, occurring prior 150°C, is a consequence of moisture loss. This is followed by a degradation with a loss of the carboxylic and hydroxyl groups in the chitosan between 170–450 °C. The thermal decomposition temperatures of the hybrid materials are around 480 °C. The results show that the hybrid films are more thermally stable than the pure polymer because of the presence of magnesium oxide. Moreover, the stability of the hybrid increases as the content of MgO increases. The mass of the remaining residue after treatment at 800 °C corresponds to the magnesium oxide content of the hybrids, indicating that the sol-gel reaction was successful.

### 3.2. Application of the CS-Mgo Nanocomposite Films As Heterogeneous Basic Catalyst

**Figure 4:**
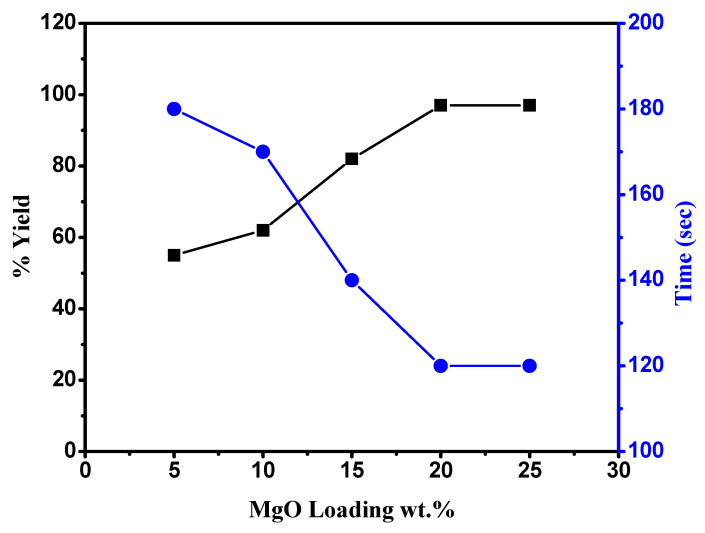
Optimization of the loading of CS-MgO film as a basic catalyst.

**Figure 5:**
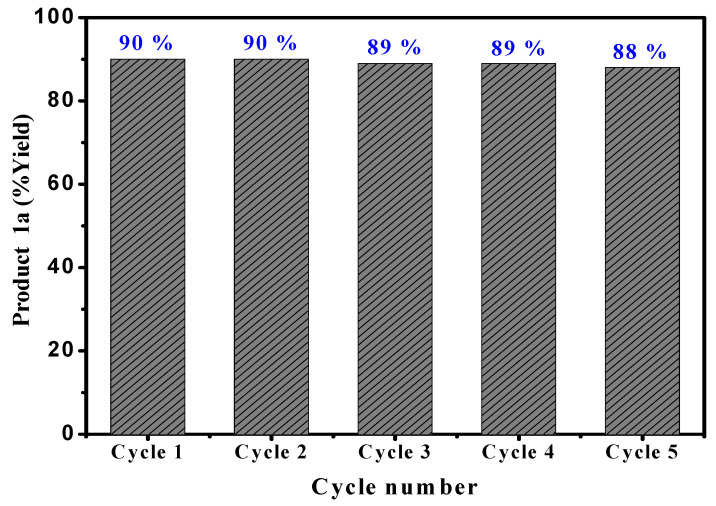
Recyclability of the CS-MgO film as a basic catalyst.

The reactions of aromatic aldehydes with guanidine hydrochloride and malononitrile, which are promoted by basic catalysts, have been found to produce pyrimidine-5-carbonitriles **1**. To investigate the catalytic activity of CS-MgO films and to optimize the reaction conditions, a preliminary study was conducted to probe the synthesis of pyrimidine derivatives through condensation reactions between benzaldehyde, guanidine hydrochloride and malononitrile in dry acetonitrile, which was promoted using different MgO loadings of the CS-MgO film catalyst (0, 5, 10, 20 and 30 wt.%). The results of this exploratory investigation demonstrate that a catalyst loading of 10 wt.% is optimal and that the process proceeds efficiently when using microwave irradiation (100 W) at 60 °C for 2 min (Figure 4). Moreover, we found that the used catalyst can be recovered; the used catalyst was thereafter subjected to an extensive washing process with hot ethanol via Soxhlet extraction for 1 h and reused four times without a significant loss of its catalytic activity (Figure 5).

**Scheme 2:**
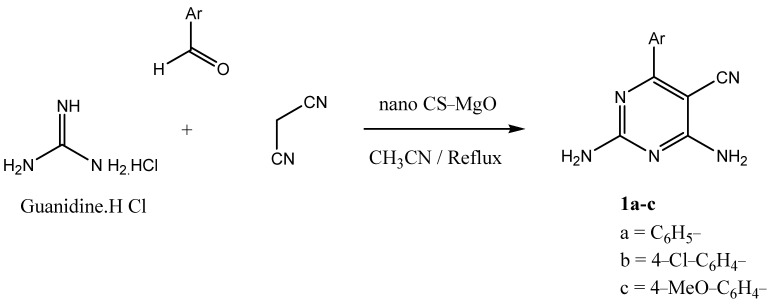
CS-MgO (10 wt.%) catalyzed reactions forming 2,4-diaminopyrimidine-5-carbonitriles.

In contrast to earlier observations [32,33], we found that the reaction promoted by 10 wt.% of CS-MgO film produced 2,4-diaminopyrimidine-5-carbonitriles **1** in an excellent yield (97%) (Scheme 2). The preliminary experiments confirmed that dry acetonitrile is the ideal solvent for the reaction. Due to the fact that the separation and contamination of products with MgO nanoparticles presents a considerable challenge, the investigated catalytic reactions were repeated under the same employed conditions in the presence of a catalytic amount of MgO nanoparticles (5 wt.%) for comparison. The results revealed that a synergistic action occurs for the combination of chitosan and MgO nanoparticles confirming that the hybrid materials work as more of a base catalyst compared to its constituent components. Thus, in a study evaluating other catalysts for this one pot reaction, stirring the mixtures of the reactants in the presence of chitosan CS powder, nano-sized MgO powder or CS-MgO film at reflux can lead to the formation of **1** in the following order of yields CS-MgO > MgO > CS.

A variety of substrate solvents of different polarity were employed to investigate the scope of the CS-MgO catalyzed one pot reaction in generating 2,4-diaminopyrimidine-5-carbonitriles (Table 1). The overall results of the investigation described above revealed that CS-MgO serves as an efficient basic catalyst for classical one-pot Knoevenagel reactions. In order to investigate the catalytic activity of the CS-MgO catalyst in the one pot synthesis of 2,6-diaminopyrimidine-5-carbonitriles and to optimize the reaction conditions, a study was conducted using different substrates, various solvents of different polarity, and under solvent free conditions (Table 1).

**Scheme 3:**
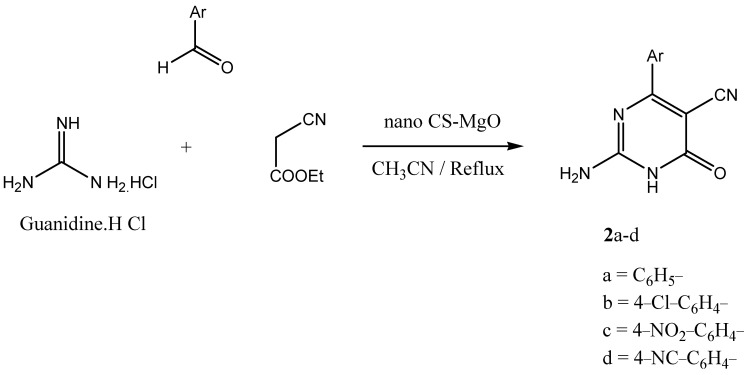
CS-MgO (10 wt.%) catalyzed reaction forming 6-oxopyrimidine-5-carbonitriles.

A similar process involving benzaldehyde, guanidine hydrochloride and ethyl cyanoacetate and the CS-MgO nano-catalyst (in comparison to the MgO nanoparticles) was observed, producing 6-oxo-pyrimidine-5-carbonitrile derivatives in relatively high yields (Scheme 3). The products of these reactions were shown to have keto structures by using NMR spectroscopy (Performed in NMR-600 Bruker) and mass spectrometry (Performed in GCms-DFS-Thermo). Although it is in disagreement with an earlier proposal [34] we found that the hydroxypyrimidine products do not exist in a solution in their enol forms. This conclusion is consistent with the observation of characteristic signals at 11.8 ppm in the ^1^H NMR spectra, which evidences the presence of lactam NH groups. Finally, a brief study was conducted to compare the catalytic activity of CS-MgO to that of MgO (Table 2). Further, the results revealed a higher catalytic effect for the hybrid nanocomposite compared with the use of MgO nanoparticles alone.

**Scheme 4:**
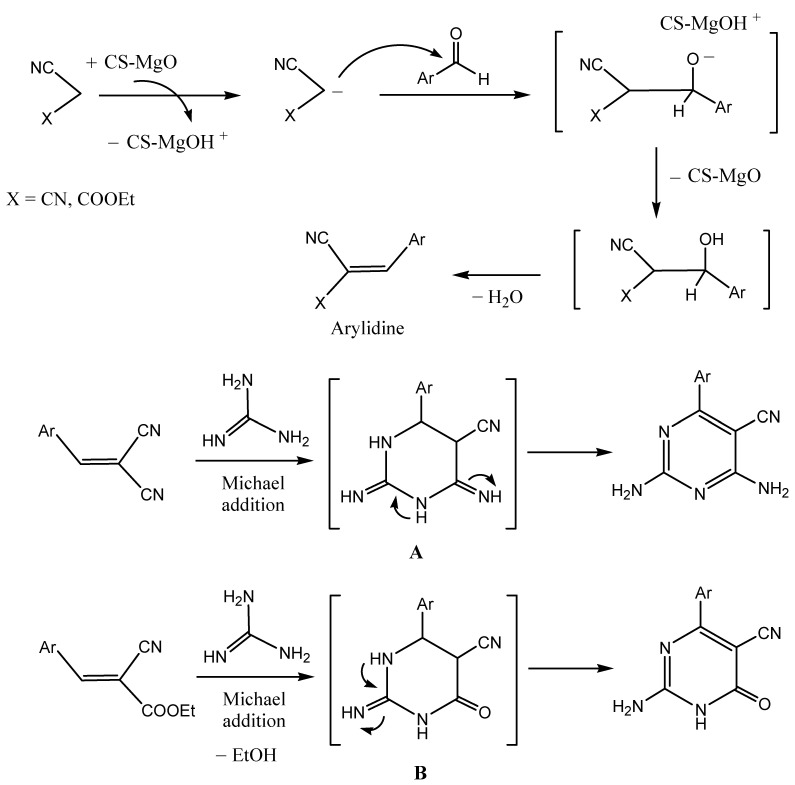
Proposed mechanism of CS-MgO (10 wt.%) catalyzed Michael addition reactions.

A plausible mechanism for the 2,4-diaminopyrimidine-5-carbonitrile forming reaction follows involves the initial generation of arylidene-malononitrile via deprotonation of malononitrile by the CS-MgO catalyst (in comparison to the MgO nanoparticles) and the addition of the enolate to the aldehyde. The arylidene-malononitrile produced in this manner undergoes a Michael addition with guanidine, which is generated from the salt precursor by proton transfer to the basic catalyst. The process ends by a route proceeding through the intermediate A or B and the generate 1a (Scheme 4).

**Scheme 5:**
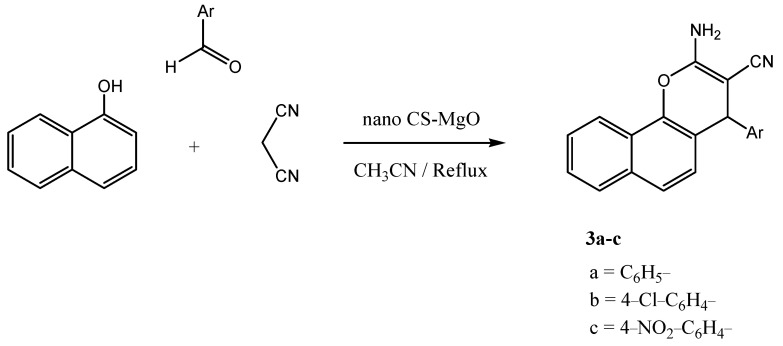
CS-MgO (10 wt.%) catalyzed reactions forming pyrimidone-5-carbonitriles.

The scope and limitations of the one-pot multicomponent reactions, promoted by the CS-MgO nano-sized catalyst (also in comparison with the MgO nanoparticles), were explored for this purpose and the multicomponent reactions of malononitrile, aromatic aldehydes, and α-naphthol were conducted using the conditions presented in Scheme 5 and Table 3. As the data in Table 3 presents, these reactions produce pyrimidone-5-carbonitriles. Again, the results presented the higher catalytic potency of the CS-MgO hybrid nanocomposite to be an excellent promoter for the investigated reactions.

**Scheme 6:**
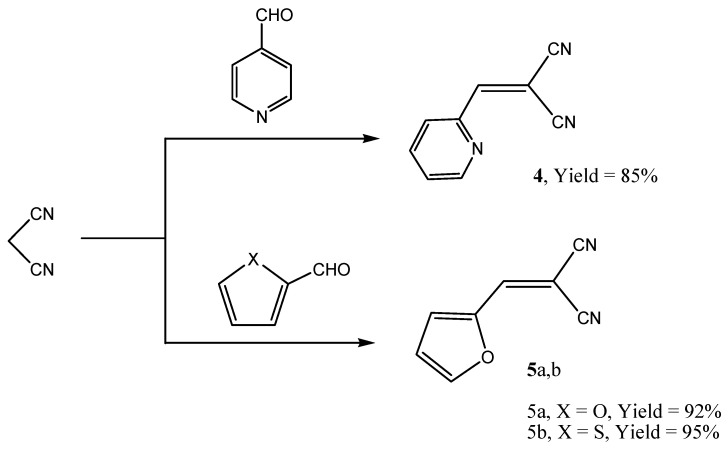
CS-MgO (10 wt.%) catalyzed reactions forming heteroaromatic arylidene-malononitrile.

Interestingly, the reaction of aromatic aldehydes, containing electron-donating substituents, occurs in relatively higher yields than those with electron-withdrawing groups. An attempt was made to utilize the CS-MgO nano-catalyst to promote arylidene-malononitrile forming reactions of pyridine-2-carboxaldehyde, furfural and thiophene-2-carboxaldehyde (Scheme 6). These reactions, conducted using malononitrile in the presence of the optimized amount (10 wt.%) of the catalyst using microwave irradiation (100 W) at 60 °C for 1 min, generated the corresponding products in higher yields (85%, 92% and 98%, respectively) than those previously reported by Bobal et al. [38] using piperidine as the base (53%, 72%, and 78% respectively). Again, it is worthwhile to mention that CS-MgO can be easily recovered from the reaction mixture and recycled up to four times without a significant loss of its catalytic activity. Also, the stability of the recovered catalyst was confirmed by comparing its IR spectra before and after the catalytic reaction.

## 4. Conclusions

Chitosan-MgO hybrid films were prepared using the sol-gel process. The results of this investigation demonstrate that a CS-Mg hybrid film was an efficient candidate as a basic, recyclable, and environmentally friendly heterogeneous catalyst for Michael addition reactions. Furthermore, preliminary studies suggested that CS-MgO (10 wt.%) nanocomposite films can be used as an efficient catalyst for Michael additions compared with bare chitosan and MgO nanoparticles. Moreover, the CS-MgO heterogeneous catalyst film can be easily recovered from the reaction mixture and can be recycled up to four times without a considerable loss of its catalytic activity. Thus, this new basic catalyst can be used as a potential alternative to other more toxic organic catalysts for Michael addition reactions such as piperidine and pyridine. In addition, the hybrid nanocomposite film presented a higher thermal stability than chitosan and, therefore, can be effectively utilized for high-temperature reactions.

## Figures and Tables

**Table 1:** Data comparing catalysts and solvent for one pot reaction forming diaminopyrimidine-5-carbonitriles (Scheme 2).

No.	Product 1	Ar	Solvent	m.p. (°C)	MgO %Yield	CS-MgO %Yield
1	1a	C_6_H_5_–	EtOH/H_2_O	234–23 [31]	70	80
2	1a	C_6_H_5_–	MeOH		78	85
3	1a	C_6_H_5_–	MeCN		-	92
4	1a	C_6_H_5_–	Solvent free		-	68
5	1b	4-Cl–C_6_H_4_–	EtOH/H_2_O	233 [31]	65	70
6	1b	4-Cl–C_6_H_4_–	MeOH		72	75
7	1b	4-Cl–C_6_H_4_–	MeCN		-	97
8	1b	4-Cl–C_6_H_4_–	Solvent free		-	78
9	1c	4-MeO–C_6_H_4_–	EtOH/H_2_O	238 [32]	72	80
10	1c	4-MeO–C_6_H_4_–	MeOH		85	90
11	1c	4-MeO–C_6_H_4_–	MeCN		-	90
12	1c	4-MeO–C_6_H_4_–	Solvent free		-	85

**Table 2:** Data comparing the catalysts and solvents for one pot reaction forming 6-oxopyrimidine-5-carbonitriles (Scheme 3).

No.	Product 2	Ar	Solvent	m.p. (°C)	MgO %Yield	CS-MgO %Yield
1	2a	C_6_H_5_–	EtOH/H_2_O	242–243	70	74
2	2a	C_6_H_5_–	MeOH		74	81
3	2a	C_6_H_5_–	MeCN		-	78
4	2a	C_6_H_5_–	Solvent free		-	69
5	2b	4-Cl–C_6_H_4_–	EtOH/H_2_O	248–250 [35]	60	62
6	2b	4-Cl–C_6_H_4_–	MeOH		66	68
7	2b	4-Cl–C_6_H_4_–	MeCN		-	69
8	2b	4-Cl–C_6_H_4_–	Solvent free		-	57
9	2c	4-NO_2_–C_6_H_4_–	EtOH/H_2_O	223–224 [34]	74	75
10	2c	4-NO_2_–C_6_H_4_–	MeOH		80	84
11	2c	4- NO_2_–C_6_H_4_–	MeCN		-	85
12	2c	4-NO_2_–C_6_H_4_–	Solvent free		-	75
13	2d	4-NC–C_6_H_4_–	EtOH/H_2_O	357–358	69	69
14	2d	4-NC–C_6_H_4_–	MeOH		78	82
15	2d	4-NC–C_6_H_4_–	MeCN		-	86
16	2d	4-NC–C_6_H_4_–	Solvent free		-	77

**Table 3:** Data comparing catalysts and solvents for one pot reactions forming f 2-amino-4-aryl-4H-benzo[h]chromene-3-carbonitrile (Scheme 5).

No.	Product 3	Ar	Solvent	m.p. (°C) obs. Lit.		MgO %Yield	CS-MgO %Yield
1	3a	C_6_H_5_–	EtOH	218	218–219 [36]	70	75
2	3a	C_6_H_5_–	EtOH/H_2_O			75	78
3	3a	C_6_H_5_–	Solvent free			-	71
4	3b	4-Cl–C_6_H_4_–	EtOH	231–232	232–233 [36]	78	84
5	3b	4-Cl–C_6_H_4_–	EtOH/H_2_O			88	92
6	3b	4-Cl–C_6_H_4_–	Solvent free			-	78
7	3c	4-NO_2_–C_6_H_4_–	EtOH	184	184–185 [37]	70	74
8	3c	4-NO_2_–C_6_H_4_–	EtOH/H_2_O			76	78
9	3c	4-NO_2_–C_6_H_4_–	Solvent free			-	67

## Data Availability

The data presented in this study are available on request from the corresponding author.
